# Supplementary material for: Antimicrobial Effects of Some Natural Products on Adhesion and Biofilm Inhibition of Clostridioides difficile
Source: Pharmaceutics. 2024 Mar 30;16(4):478. doi: 10.3390/pharmaceutics16040478 (PMC11054867; doi:10.3390/pharmaceutics16040478)
Supplement: Supplementary file 1 [file pharmaceutics-16-00478-s001.zip › pharmaceutics-2905714-supplementary.pdf]

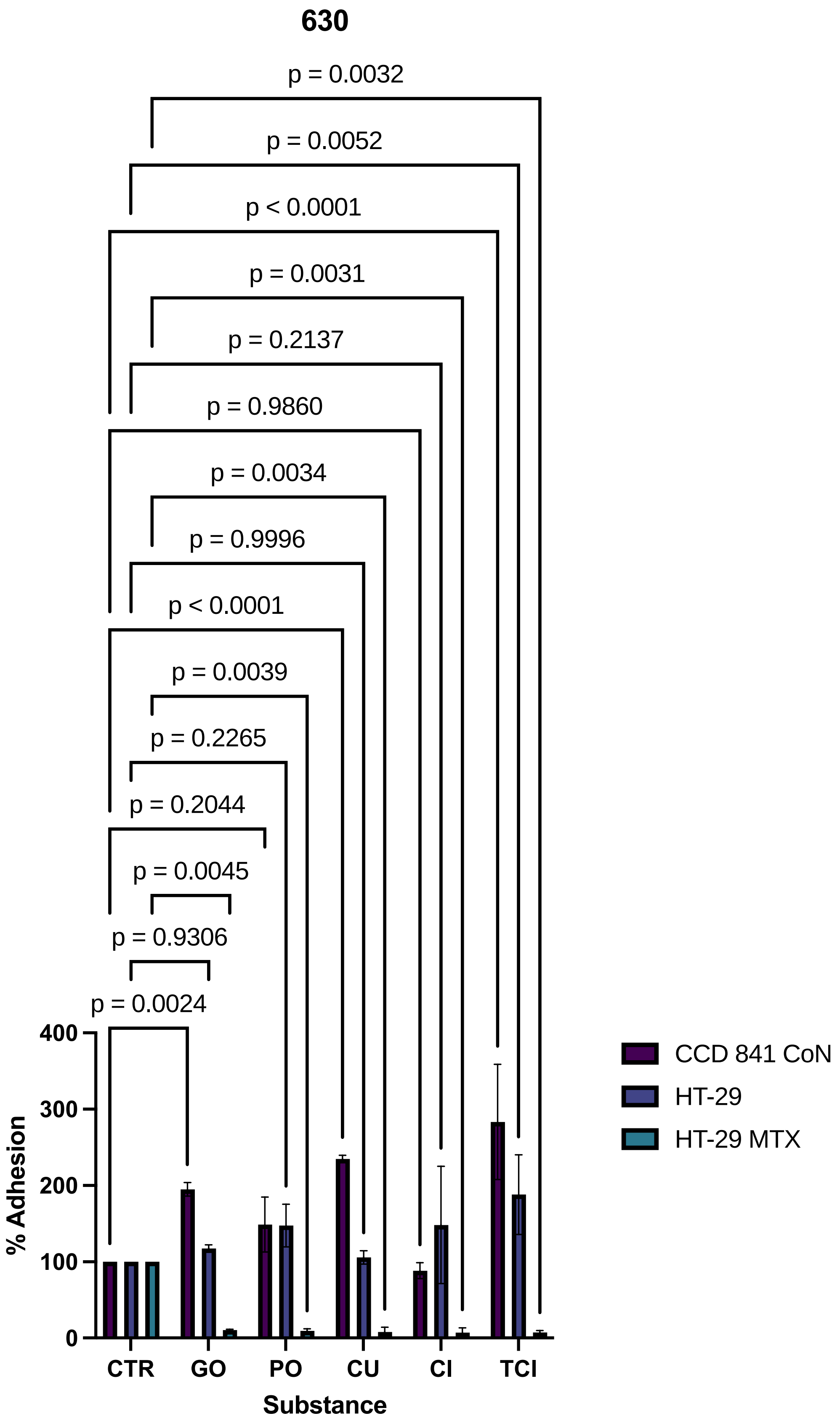

Figure S1. The effects of tested substances on adhesion of *C. difficile* strain 630 to three different cell lines including pairwise comparisons. Error bars represent standard deviations. CTR – positive control; GO – ginger oil; PO – peppermint oil; CU – curcumin; CI – cinnamaldehyde; TCI - trans-cinnamaldehyde.

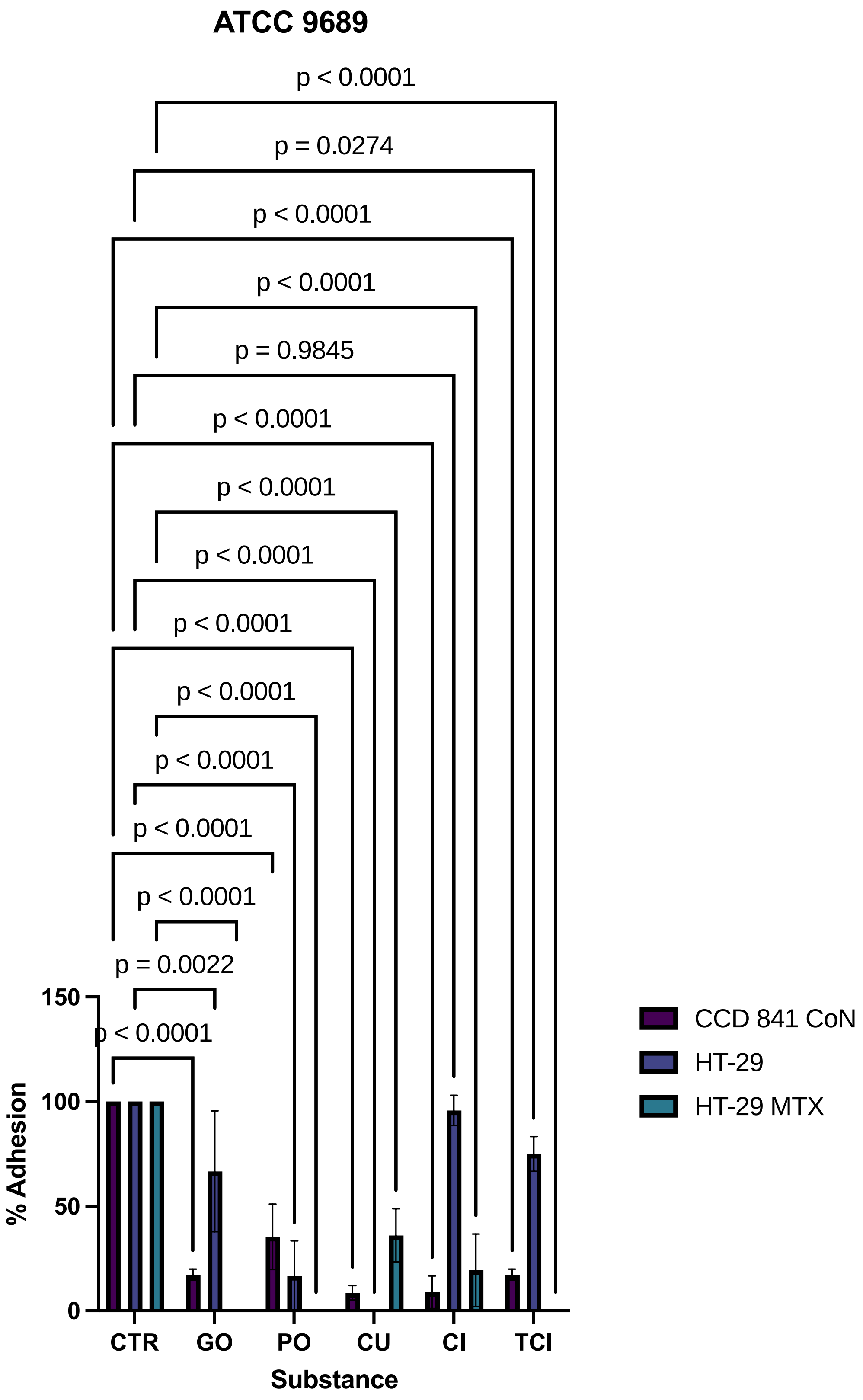

Figure S2. The effects of tested substances on adhesion of *C. difficile* strain ATCC 9689 to three different cell lines including pairwise comparisons. Error bars represent standard deviations. CTR – positive control; GO – ginger oil; PO – peppermint oil; CU – curcumin; CI – cinnamaldehyde; TCI - trans-cinnamaldehyde.

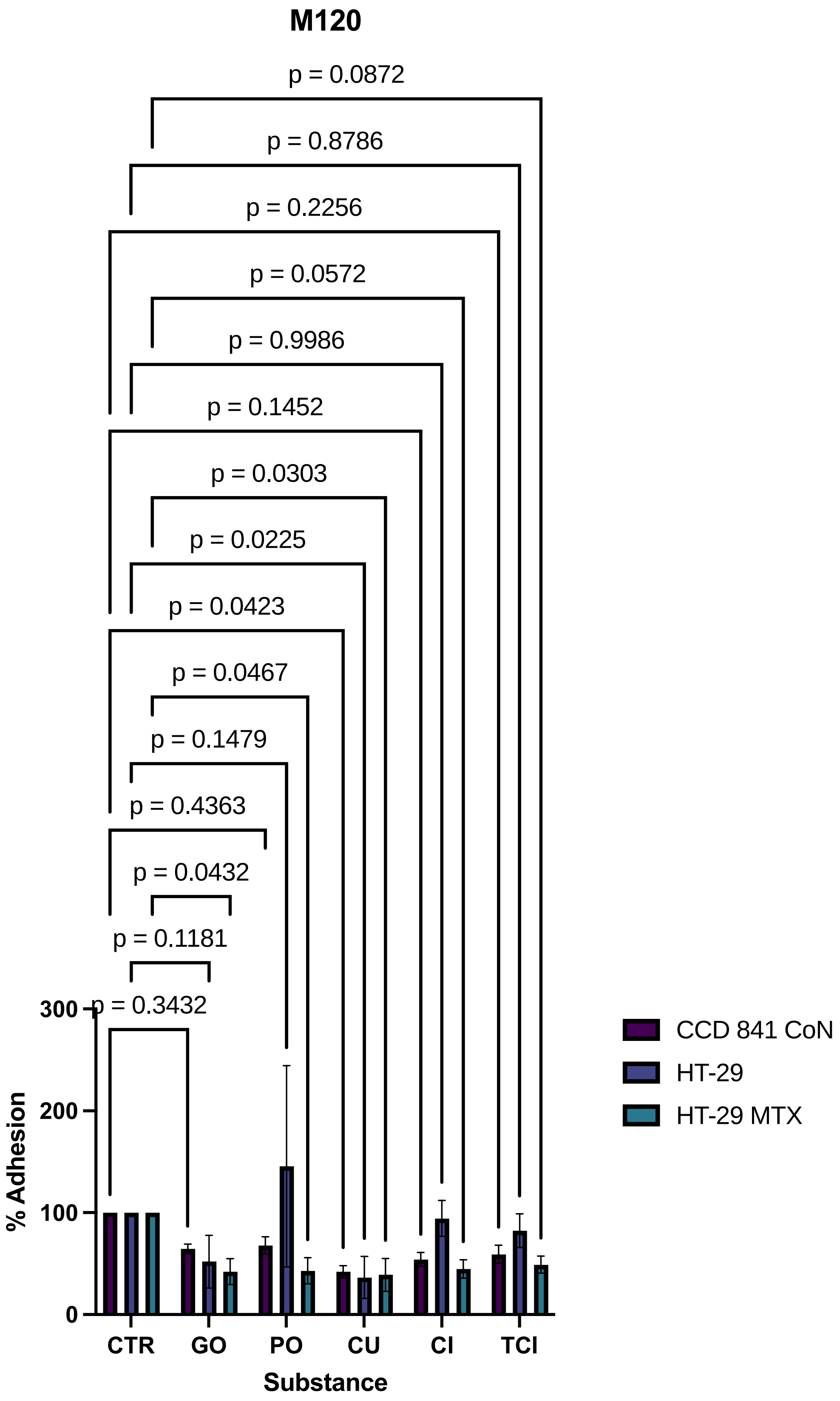

Figure S3. The effects of tested substances on adhesion of *C. difficile* strain M120 to three different cell lines including pairwise comparisons. Error bars represent standard deviations. CTR – positive control; GO – ginger oil; PO – peppermint oil; CU – curcumin; CI – cinnamaldehyde; TCI - trans-cinnamaldehyde.

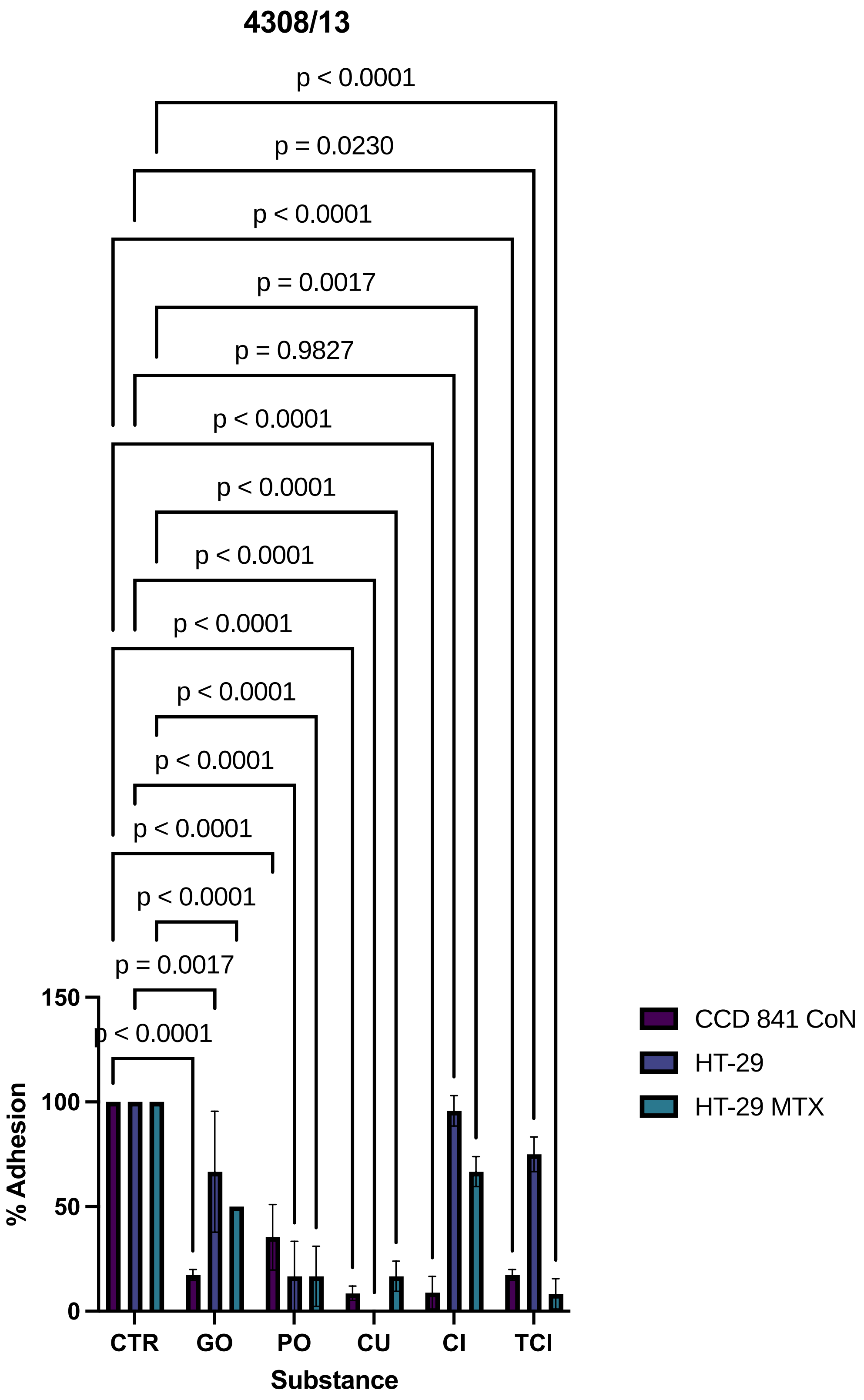

Figure S4. The effects of tested substances on adhesion of *C. difficile* strain 4308/13 to three different cell lines including pairwise comparisons. Error bars represent standard deviations. CTR – positive control; GO – ginger oil; PO – peppermint oil; CU – curcumin; CI – cinnamaldehyde; TCI - trans-cinnamaldehyde.

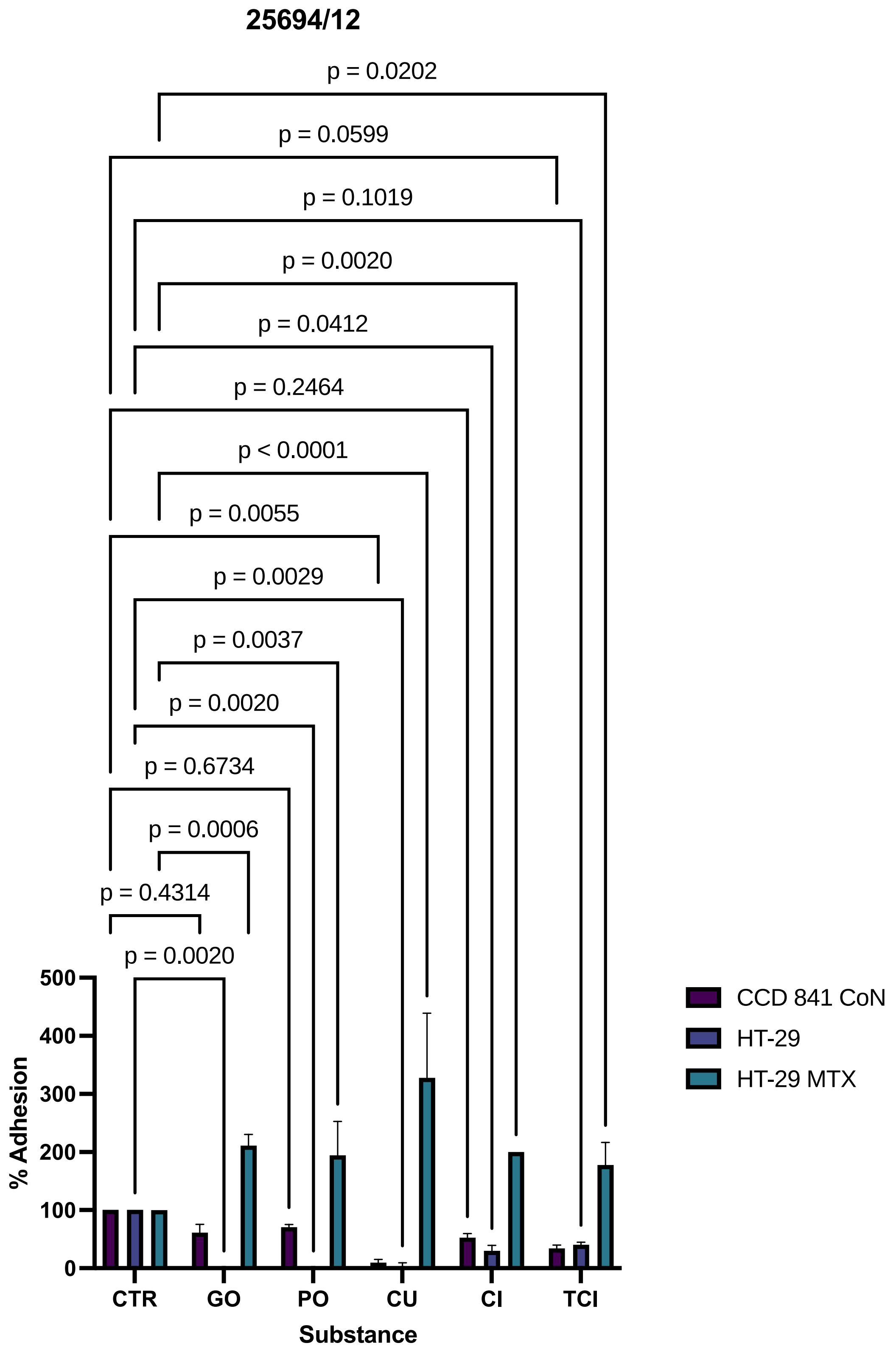

Figure S5. The effects of tested substances on adhesion of *C. difficile* strain 25694/12 to three different cell lines including pairwise comparisons. Error bars represent standard deviations. CTR – positive control; GO – ginger oil; PO – peppermint oil; CU – curcumin; CI – cinnamaldehyde; TCI - trans-cinnamaldehyde.

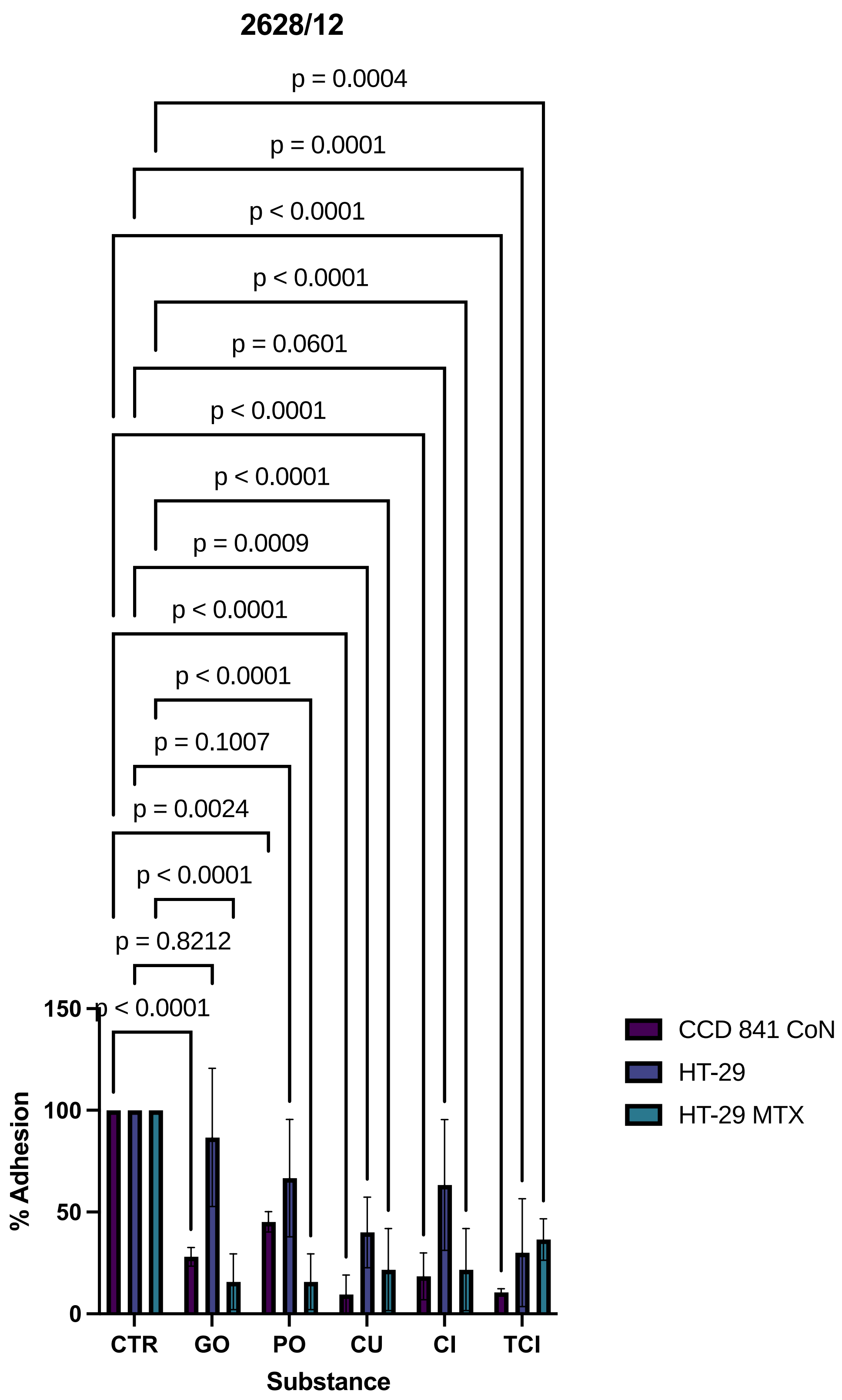

Figure S6. The effects of tested substances on adhesion of *C. difficile* strain 2628/12 to three different cell lines including pairwise comparisons. Error bars represent standard deviations. CTR – positive control; GO – ginger oil; PO – peppermint oil; CU – curcumin; CI – cinnamaldehyde; TCI - trans-cinnamaldehyde.

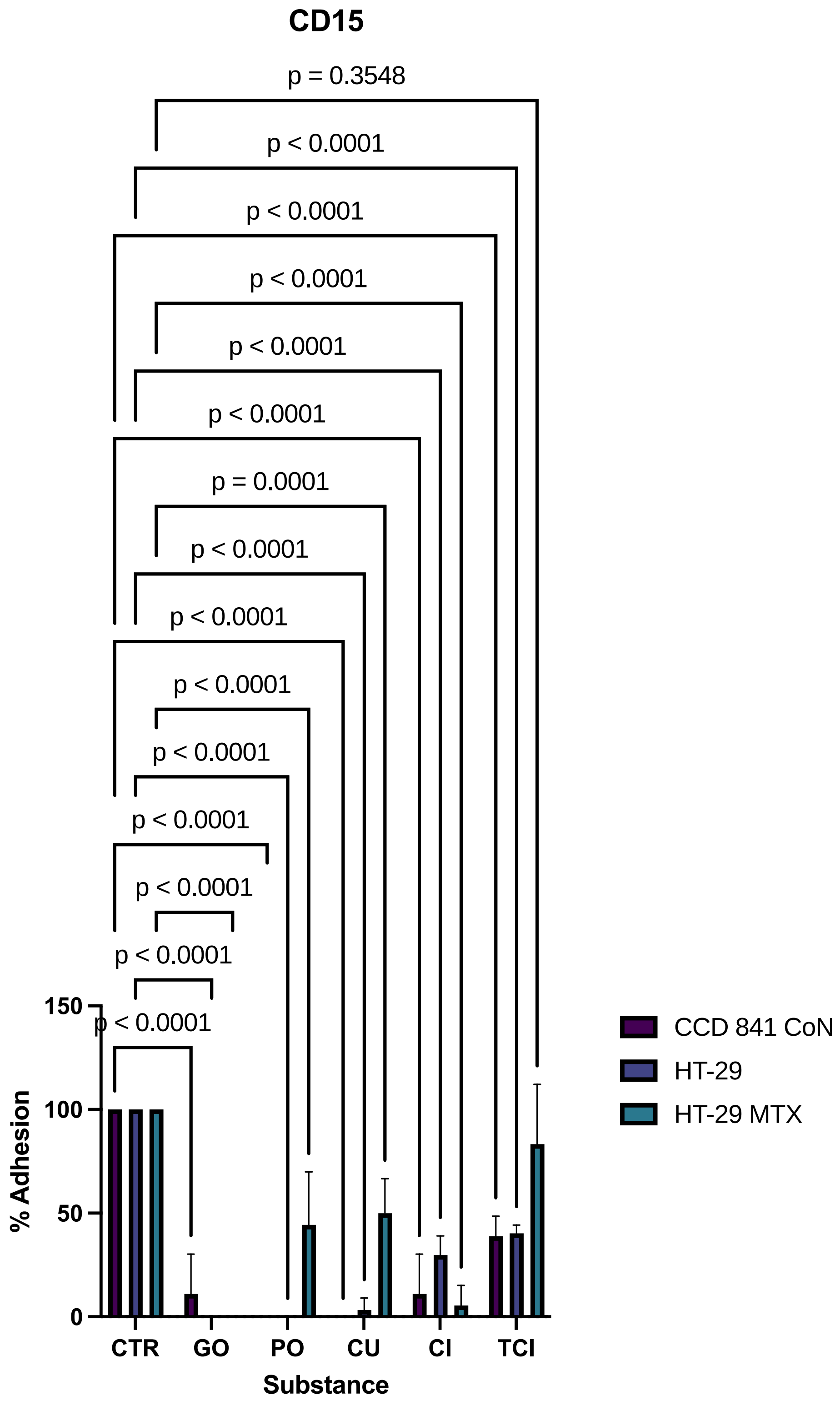

Figure S7. The effects of tested substances on adhesion of *C. difficile* strain CD15 to three different cell lines including pairwise comparisons. Error bars represent standard deviations. CTR – positive control; GO – ginger oil; PO – peppermint oil; CU – curcumin; CI – cinnamaldehyde; TCI - trans-cinnamaldehyde.

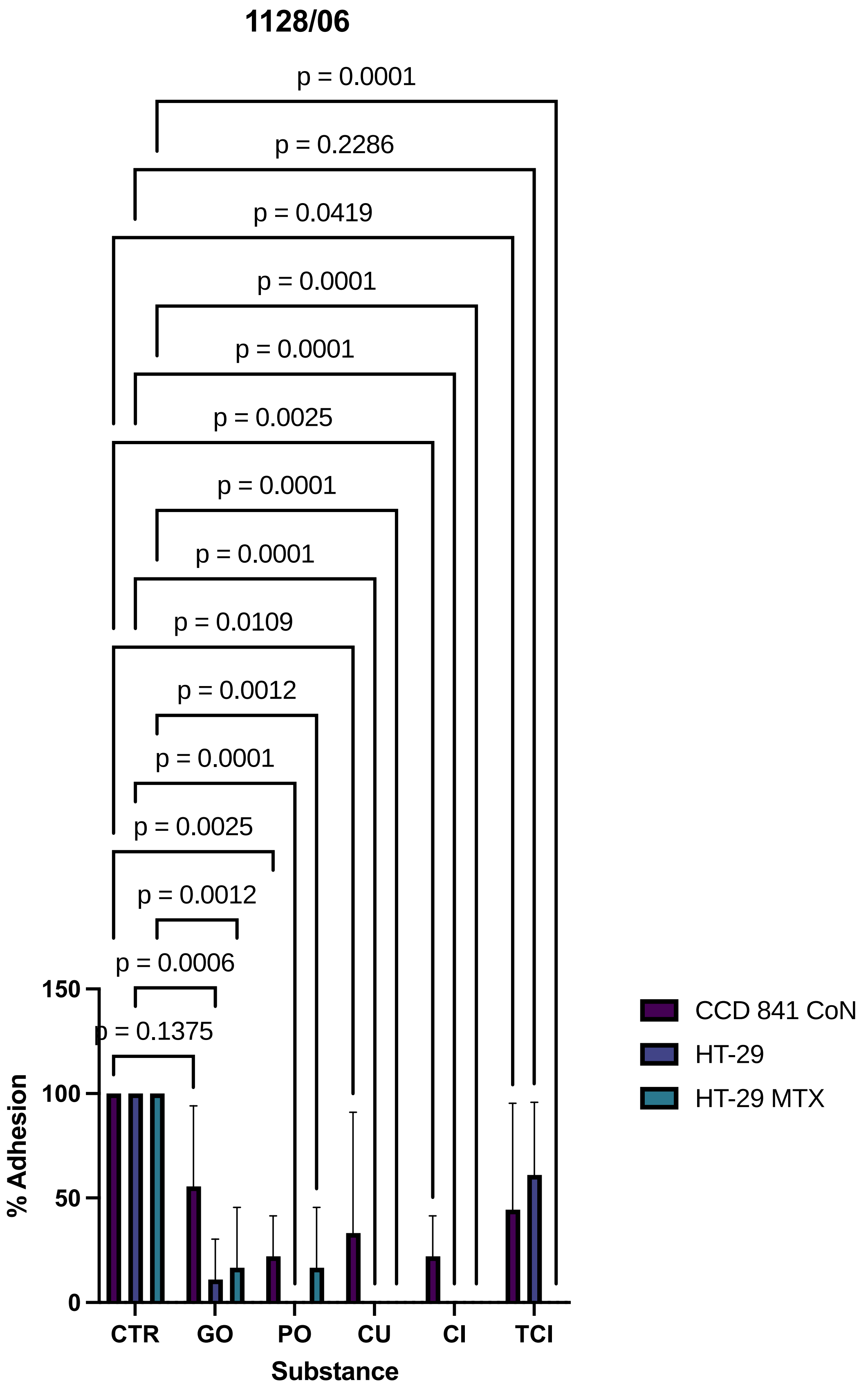

Figure S8. The effects of tested substances on adhesion of *C. difficile* strain 1128/06 to three different cell lines including pairwise comparisons. Error bars represent standard deviations. CTR – positive control; GO – ginger oil; PO – peppermint oil; CU – curcumin; CI – cinnamaldehyde; TCI - trans-cinnamaldehyde.
